# Clinical symptoms and faecal shedding of SARS-CoV-2 RNA among hospitalized COVID-19 patients: Implication for transmission

**DOI:** 10.1371/journal.pgph.0003571

**Published:** 2024-08-28

**Authors:** Rebecca Folasade Bamidele, Adeola Fowotade, Rasheed Bakare, Olufunso Opeyemi Bamidele, Chukwuma Ewean Omoruyi, Amos Abimbola Oladunni, Temitope Alonge

**Affiliations:** 1 Department of Medical Microbiology and Parasitology, University College Hospital, Ibadan, Oyo state, Nigeria; 2 Biorepository Laboratory, College of Medicine, University College Hospital, Ibadan, Oyo state, Nigeria; 3 Department of Community Medicine, University College Hospital, Ibadan, Oyo state, Nigeria; 4 Department of Pharmacology and Therapeutics, College of Medicine, University of Ibadan, Ibadan, Oyo state, Nigeria; 5 Department of Orthopedic Surgery, University College Hospital, Ibadan, Oyo state, Nigeria; University of St. Andrews, UNITED KINGDOM OF GREAT BRITAIN AND NORTHERN IRELAND

## Abstract

SARS-CoV-2 shedding in human stool has been suggested as a probable route for faeco-oral transmission of the virus due to the availing evidence on the infectivity and pathogenicity of similar highly infectious respiratory viruses. Determining association of SARS-CoV-2 shedding in stools and presenting clinical status might be useful for prediction of the viral transmission spectrum and disease outcome. This study involved a descriptive cross-sectional survey of 91 consenting hospitalized, confirmed COVID-19 patients in Infectious Disease Isolation Centre, Oyo State, Nigeria. Socio-demographic characteristics and other ancillary data were collected from patient’s hospital records with the aid of a structured investigator administered questionnaire. The laboratory detection of SARS-CoV-2 RNA in the stool of patients was performed using RT-PCR method. 27 (29.7%) of the 91 COVID-19 patients shed SARS-CoV-2 in their stools. The frequency of male (38.3%) patients shedding the virus in stools was higher than female (12.9%) patients (P = 0.012). Higher proportion of patients who had symptoms (41.2%) at admission shed the virus in their stool (P <0.007); particularly, fever (0.001), fatigue (0.003), headache (0.003), catarrh (0.001), and loss of smell (0.009). The frequency of viral shedding in stool was higher among patients with loss of taste (p = 0.028). Viral shedding in stool was significantly associated with low CT values (47.2%) and moderate CT value (21.4%) (P<0.05). Multivariate analysis showed that patients with moderate CT-value (OR = 0.28, 95% CI: 0.08–0.94, P = 0.039) and high CT-value (OR = 0.08, 95% CI: 0.01–0.80, P = 0.033) were less likely to shed the virus in stool. The gastrointestinal tract could be a route of SARS-CoV-2 transmission irrespective of the patients’ clinical status. The low and moderate CT values of the nasopharyngeal swab is associated with shedding of the virus in patients’ stools, although infectivity will depend on viral activity obtainable from further laboratory test analyses, such as viral culture.

## Introduction

Besides the common respiratory symptoms, COVID-19 is also associated with multisystem manifestations including involvement of the gastrointestinal system [1]. Many members of the Coronaviridae family are zoonotic, and characteristically infect both the respiratory and/or gastrointestinal tracts of mammals, including humans [2]. Consequently, there are emerging evidence of SARS-CoV-2 shedding in several body fluids, including sputum, urine, throat swab, and stool samples in symptomatic and asymptomatic patients [3]. A significant percentage of COVID-19 patients have reportedly resented with varying gastrointestinal symptoms, including diarrhoea, nausea, vomiting, abdominal discomfort even before the development of respiratory symptoms. The variation in shedding sites for the SARS-CoV-2 present a cautionary probability to the multiple transmission routes of the virus [4].

Hospitalized COVID-19 patients are usually symptomatic or asymptomatic at the time of presentation. Mild symptomatic patients often progress to pneumonia some of which proceed to severe respiratory illness that will sometimes require intensive care unit management amidst the emergence of some complications [5]. Common symptoms of COVID-19 include fever, cough, sneezing, shortness of breath, myalgia, fatigue, loss of taste and/or smell and hiccups. Complications include acute respiratory distress syndrome, lung paralysis and death [6].

In many instances, the severity of the disease is often associated with the presenting clinical states, including the co-morbid states of hospitalized patients. In a review of clinical and laboratory data of the early cases of COVID-19 in Europe, three distinct presenting clinical states were reported. These were: a pauci-symptomatic state, a progressive disease state; and a persistently critical state, involving multiple organ failures [3].

## Materials and methods

### Study design, setting and period

This is a descriptive cross-sectional study of stool samples collected from hospitalized confirmed COVID-19 patients, for shedding of SARS-CoV-2 in their stool. The study was conducted in the Infectious Disease Centre (IDC) at Olodo, Ibadan, Oyo State, Nigeria. IDC Olodo is the State’s major isolation centre for patients with COVID-19. Other Infectious Disease Centres (IDC) within the State are the 20-bedded University College Hospital Ibadan; and newly constructed IDCs at Oyo, Saki and Iseyin. The IDC, Olodo, Ibadan has managed more than 96% of hospitalized COVID-19 patients in the state. The Infectious Disease Centre, Olodo, Ibadan is located in Egbeda Local Government Area (LGA) of the State; which is a semi-urban settlement of the capital city of Ibadan. The Isolation Centre is about a hundred bedded, consisting of 10 beds in the Intensive Care Unit (ICU), 12 beds in the High Dependent Unit, 22 beds in the male General Ward, 12 beds in the female General Ward, and about 42 beds in six other side wards. According to the 2006 census, Ibadan is the largest city in sub Saharan Africa, divided into urban and semi-urban areas measuring 3, 080km2 (1,190sq miles) and 6,800km2 (2,600sq miles) respectively. It is lying between latitude 7.401962 and longitude 3.917313 with GPS coordinates of 7 24’ 7.0632” N and 3 55’ 2.3268” E [7].

### Study population

The study population were PCR, confirmed COVID-19 patients, admitted at the Infectious Disease Centre at Olodo, Ibadan.

### Sample size determination

Sample size was calculated using the formula for a single proportion study. For this study, the total minimum sample size is 88 stool samples.

### Sampling strategy

Consenting admitted patients to the Isolation Centre were consecutively recruited into the study until the estimated sample size was reached. The study subjects were recruited and this was over a period of three months.

### Inclusion and exclusion criteria

Inclusion criteria were consented patients with PCR confirmed COVID-19 test for SARS-CoV-2 admitted at the Oyo State Infectious Disease Centre and age ≥ 18 years.

Exclusion criteria included patients with moderate to severe form of disease, individuals less than 18 years of age, pregnant and lactating mothers.

### Specimen collection

Stool samples were collected from patient at admission after taking written informed consent from them. About 20 grams of stool sample was collected from each patient, into a wide mouth universal bottle and the samples were temporarily stored at -20°C for less than 24 hours at the isolation centers. The samples were then transferred in a cold chain at -80°C to the Biorepository Laboratory where they were stored at -80°C before final processing.

### Data collection procedure

Data were collected with the aid of a structured self/investigator-administered questionnaire and from ancillary information obtained from the patient’s hospital records. The ancillary information included clinical and laboratory parameters of the selected participants. The questionnaire was used to collect sociodemographic data, clinical presentation spectrum of COVID 19 and risk factors. The data were sorted out using Statistical Package for Social Sciences (SPSS), and stored on the computer using Microsoft Office Excel. The questionnaires were designed in English language, translated to Yoruba, and then back-translated to English, in order to maintain the original meaning of the instruments. Data were collected over 3-month duration between 5^th^ of May 2021 and 5^th^ of July 2021. In view of the infective potentials of the virus, three of the nurses working in the Isolation Centre were recruited as research assistants. They were properly briefed and trained to assist in stool sample collection and questionnaire administration for the selected participants. A written informed consent were obtained from participants before recruitment into the study and patients’ identity were kept anonymous.

### Laboratory procedure

#### Sample collection, transportation and storage

A sterile wide-bore universal sample bottle was used to collect stool sample from each patient in order to maintain the RNA in a stable state. The samples were immediately temporarily stored in a Geostyle box with ice packs, at -20°C and then transported into a minus 80 degrees (-80°C) freezer in the laboratory till further processing.

#### Biosafety measures

The laboratory diagnosis of SARS-CoV-2 in the stool of patients was undertaken at the Biorepository Clinical Virology Laboratory, Ibadan. All biosafety instructions including the use of personal protective equipment appropriate for standard, contact and air borne precautions were strictly followed. Proper hand hygiene, use of gowns, respirator (N95 or FFP2), eye goggle or face shield and gloves were adopted.

#### Stool sample preparation

At the analytical stage, the stool samples were treated by adding about 5mL of phosphate buffer solution (PBS) into a centrifuge tube containing glass beads (Vendor: Machery-Nagel’s beads for bio-analysis; Cat. Ref No.: 740786.B.250) in order to lyse the stool samples. This was followed by addition of 1g of stool sample using an applicator stick into the centrifuge tube. The tube was vortexed vigorously and centrifuged at 2415g for 10min. The supernatant fluid was then removed into two cryovials and stored at -80 degrees while the sediment in the centrifuge tube was discarded.

#### RNA extraction

RNA extraction was done using standard extraction protocols/kits (DaAn GENE., Guangdong, P.R. China). 200uL of treated sample was added into a centrifuge tube and equal volume of lysate working solution containing carrier RNA was added to the tube. This was vortexed for 15 seconds for homogenization. The mixture was then incubated for 10 minutes after which 250uL of ethanol was added and vortexed for another 15 seconds. The whole mixture was centrifuged at 1774g followed by addition of 500uL of inhibitor and centrifuged again at 1774g. The spin column was changed into a new collecting tube, placed at room temperature and centrifuge at 2415g for 3min to remove any residual ethanol. A volume of 50uL preheated eluent at 72 degrees (72°C) was added, incubated for 1 minute at room temperature and centrifuged at 2415g for 1 minute.

#### Real-Time PCR

Real-time Reverse Transcriptase-Polymerase Chain Reaction (rRT-PCR) assays was employed as the molecular diagnostic test for the SARS-CoV-2 isolates (S1 Text). In the post-analytical stage, the molecular findings were carefully interpreted in relation with the hospitalized patients’ socio-demographic status and presenting clinical states. Appropriate measures were taken to protect laboratory staff while maintaining standard laboratory operating procedure.

#### Procedure for rRT-PCR

The rRT-PCR was carried out using the Invitrogen Superscript III Platinum One-Step qRT-PCR system (ref: 11732–088) which targets nCoV_IP2 / 12621–12727, nCoV_IP4 / 14010–14116 and E gene / 26141–26253 according to manufacturer instructions. The PCR was carried out in a final reaction volume of 25μl containing 20μL of master mix and 5μL of extracted RNA. The amplification condition consists of initial reverse transcription at 55°C, for 20 minutes, denaturation at 95°C for 3 minutes, forty five (45) cycles of 95°C for 15 seconds, 55°C for 45 seconds and 72°C for 15 seconds. Positive, negative, internal and negative extraction (NEC) controls were included in each run.

After amplification, the PCR products were viewed on the computer screen, presence of an S-shaped amplification curve to the target gene was interpreted as positive while absence of the amplification curve to the two target genes (N and RdRp genes) was recorded as negative.

### Quality control for rRT-PCR

#### No template control

A no template control (NTC) was included in each rRT-PCR run. The NTC underwent the extraction process in the absence of a sample. The NTC enables detection of contamination of the extraction process and PCR reagents. Detection of a positive result in a NTC reaction indicates the presence of contaminating nucleic acid.

#### Positive control

The rRT-PCR kit contained a positive control which was included in each run. A positive result will indicate that the PCR reagents were in optimal condition and effective.

#### Negative control

A negative control (RNase free water) will be included in each run. A negative result indicated that there was no reagent contamination.

### Data analysis

All collected data (S1 Data) were daily checked and cleaned of all errors. Missing data were duly sorted-out before the data were properly entered into the computer. Analysis were done using the Statistical Package for Social Sciences Version 25 (IBM SPSS Version 25).

Results were presented in form of frequencies, percentages, tables, proportions, graphs, and charts; wherever applicable. Frequencies and percentages were presented for categorical variables of interest. Variables were summarized using means, medians, standard deviations and interquartile ranges. Univariate analysis was done by generating frequencies of the variables. Bivariate associations were tested using inferential statistics. Chi-square test was used to establish associations between categorical variables. Continuous variables were tested against nominal variables using one-way analysis of variance (ANOVA). Independent variables that are significant at 5% level of significance or that were significant from the review of literature were included in the multivariate analysis. Logistic regression was used to test their associations with the dependent variables by cross-tabulating the dependent variables with the independent variables.

### Dependent variable

SARS-CoV-2 Shedding in Stool Samples.

### Independent variables

Presenting Clinical States, presence of co-morbid states and socio-demographic characteristics (Age, Sex, Place of residence, Occupation).

### Ethics

A written ethical approval letter was obtained from Oyo state Ministry of Health Ethical Review Board.

## Results

### Socio-demographic characteristics of respondents

Table 1 shows the socio-demographic characteristics of the respondents. The mean age of the patients was 37.78±14.41years. About 40% of the patients are in the age group 25–34 years, 65.9% are males, Yoruba tribe and 84.6% are Christian by religion. More than half of the patients are from the non-slum. Many (60.4%) of the patients are civil servants.

**Table 1:** Frequency distribution of socio-demographic characteristics of participants.

Variables	Number	Percentage
**Age (Years)**		
≤24	11	12.1
25–34	37	40.7
35–44	21	23.1
≥45	22	24.2
Mean ± SD	37.78±14.41	
**Sex**		
Male	60	65.9
Female	31	34.1
**Ethnicity**		
Yoruba	67	73.6
Hausa	5	5.5
Igbo	17	18.7
Others	2	2.2
**Religion**		
Christian	77	84.6
Islam	14	15.4
**Residence**		
Semi-slum	43	47.3
Non-slum	48	52.7
**Occupation**		
Healthcare Workers	13	14.3
Civil Servants	20	22.0
Customer Service Workers	23	25.2
Military/Armed Forces	6	6.6
Traders & Unskilled workers	13	14.3
Unemployed	16	17.6

### Clinical parameters of the respondents

Table 2 also shows the mean CT score of the patients’ nasopharyngeal samples on rRT-PCR is 30.94±5.90 and that of stool sample is 33.14±2.74. 27 (29.7%) out of 91 patients shed the virus in their stools.

**Table 2:** Frequency distribution of SARS-CoV-2 detection from laboratory parameters of patients.

SARS-CoV-2 Detection	Frequency	Percentage
**Virus Shedding Status**		
Positive	27	29.7
Negative	64	70.3
Total	91	100
**Mean CT**		
Stool Sample	33.14 +/- 2.74	
NP	30.94 +/- 5.90	

Table 3 shows the clinical parameters of the respondents. More than half of the respondents had symptoms at admission. About 33% of these symptomatic respondents had fever, 29.7% had fatigue while 36.3% had headache.

**Table 3:** Clinical presentation profile of participants.

Variables	Yes (%)	No (%)
Symptoms on Admission	51 (56%)	40 (44%)
Fever	30 (33%)	61 (67%)
Fatigue/Body weakness	27 (29.7%)	64 (70.3%)
Headaches	33 (36.3%)	58 (63.7%)

**Fig 1:**
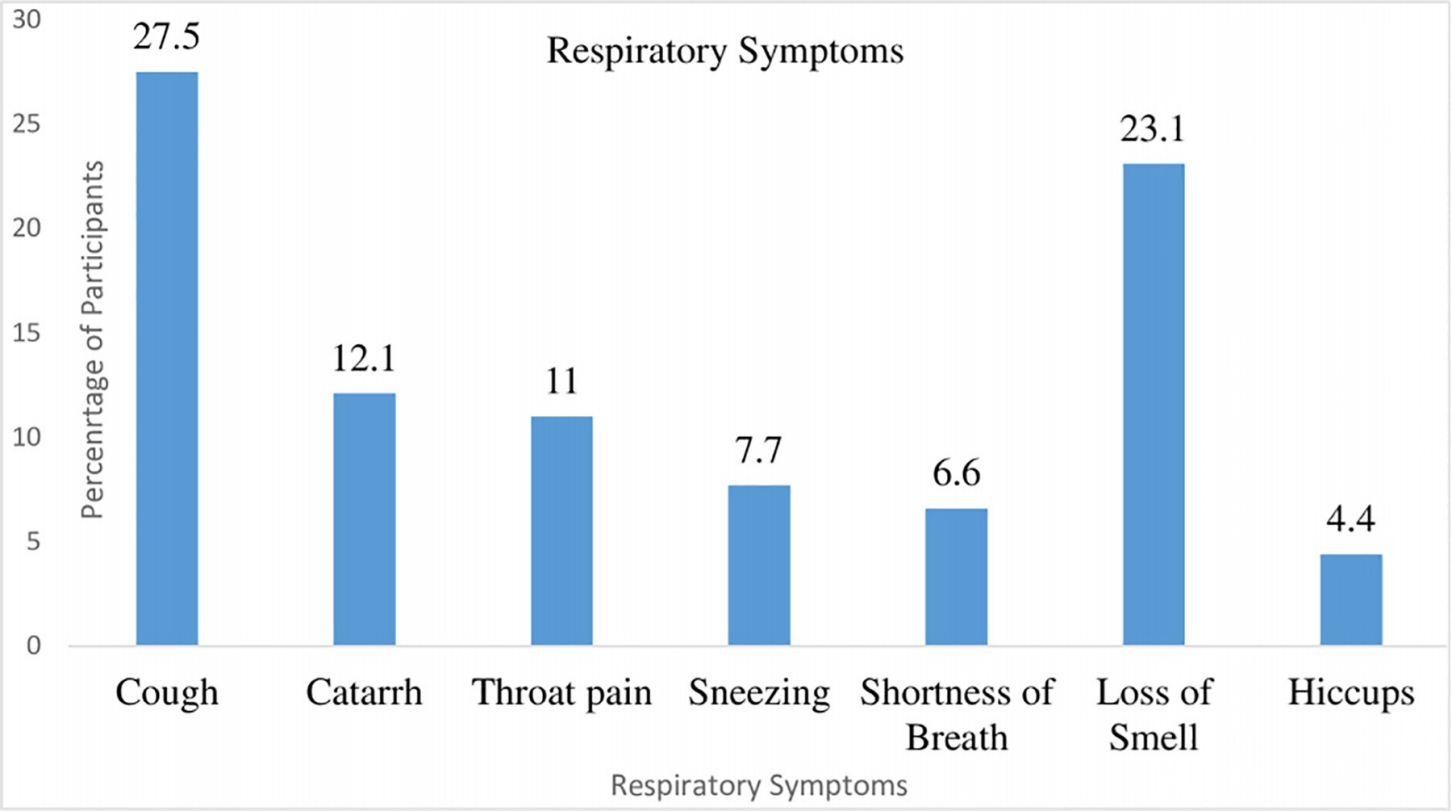
Respiratory symptoms of the participants.

About 28% of the respondents had a cough as the respiratory symptoms at the point of admission, Loss of smell (23.1%), Catarrh (12.1%), Throat pain (11%), Sneezing (7.7%) Shortness of breath (6.6%) and hiccups (4.4%) **Fig 1.**

**Fig 2:**
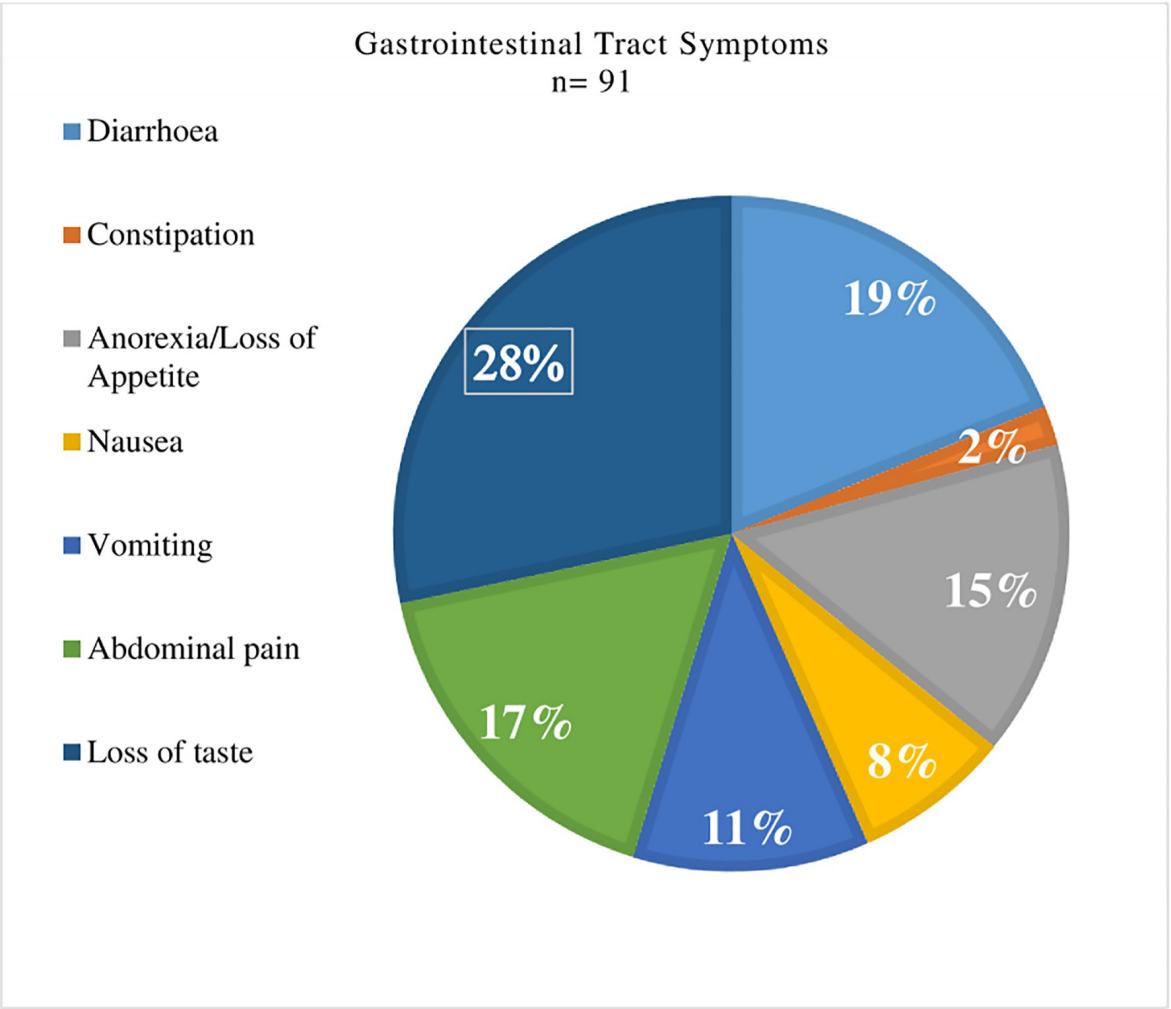
Gastrointestinal tract symptoms of the participants.

At admission, about 28% of the respondents had Loss of taste, 19% had Diarrhoea, Abdominal pain (17%), Anorexia (15%), Vomiting (11%), Nausea (8%) and Constipation (2%) **Fig 2.**

**Fig 3:**
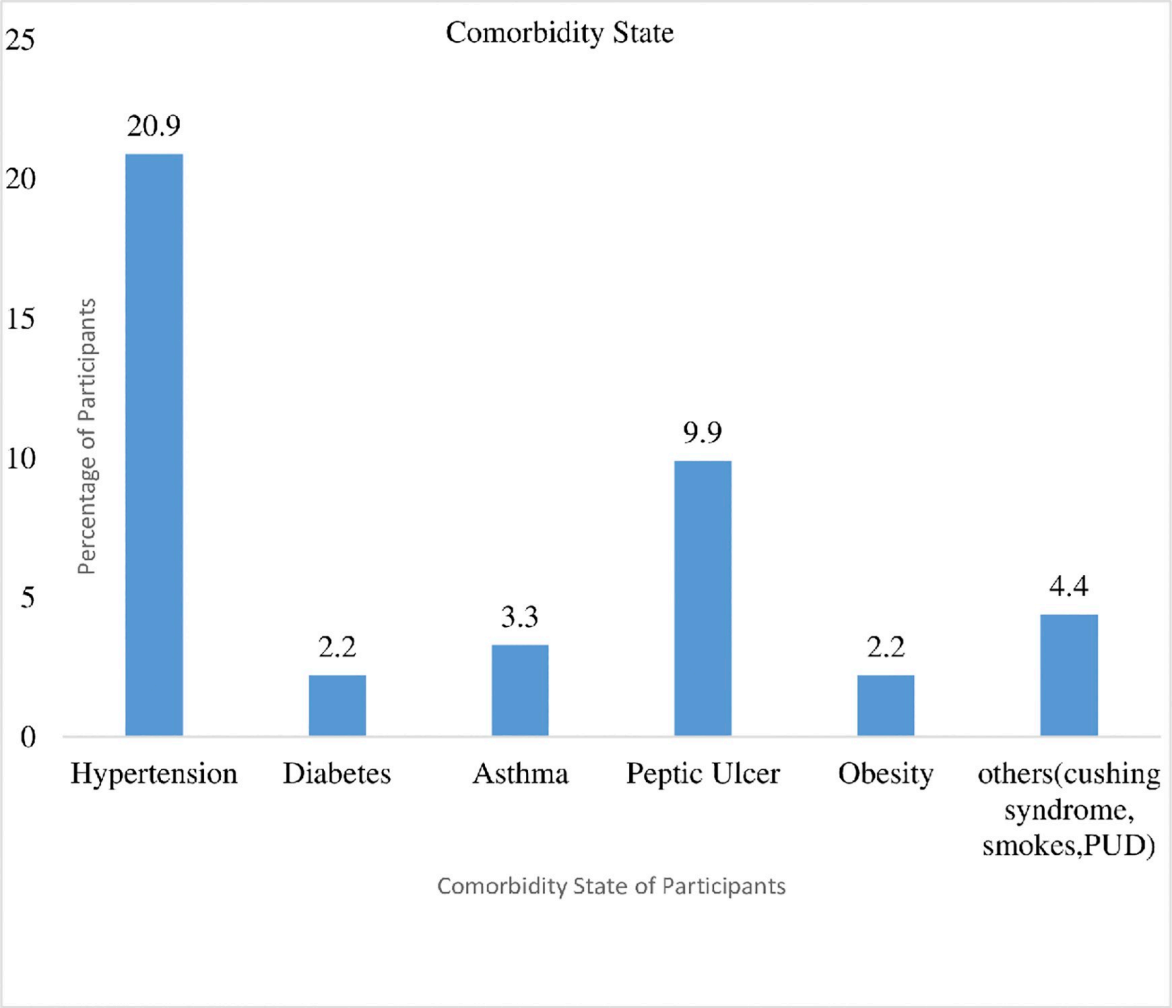
Comorbidity state of the participants.

About 21% of the participants had hypertension at the point of admission, Peptic Ulcer (9.9%), Asthma (3.3%) Obesity, Diabetes (2.2%) **Fig 3.**

**Fig 4:**
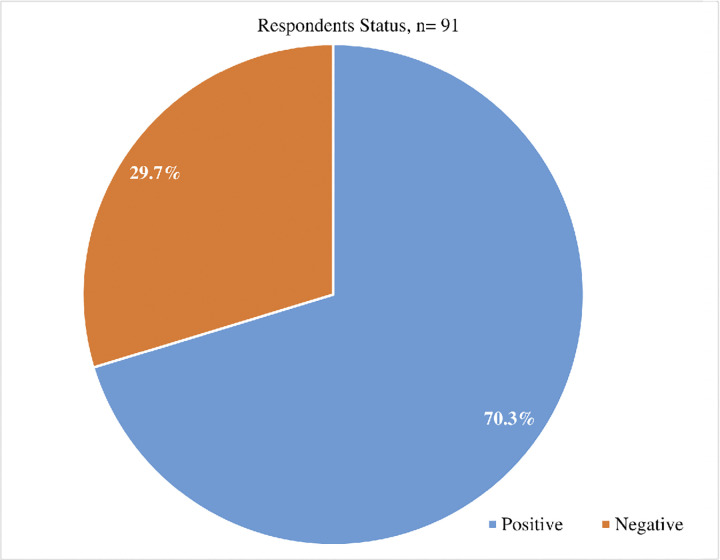
Participants status on the stool shedding.

Approximately 70% of the participants were positive of the viral stool shedding compared to 30% that were negative **Fig 4.**

### Association between socio-demographic characteristics and SARS-CoV-2 shedding in stools

Table 4 shows the association between socio-demographic characteristics and SARS-CoV-2 shedding in Stools. The proportion of respondents that were positive for shedding SARS-CoV-2 in stools were highest among those between the age group of 35–44 years (42.9%) compared with those who were ≥45years (36.4%), 25–34 (24.3%) and ≤24years (9.1%) at P-value >0.05. The proportion of male (38.3%) participants that were shedding the virus in stools was higher compared with female respondents (12.9%) at P-value<0.05. The association of occupation with the shedding of SARS-CoV-2 is not significant, at p > 0.05; where Military/Armed Forces respondents (50%) had the highest positive status, followed by the civil servants (45%), health care workers (38.5%), traders (30.8%), unemployed (18.8%), with customer care workers (13%) being the lowest. However, it is important to note that the SARS-CoV-2 shedding in Military respondents was equal for both positive and negative status, at 50%.

**Table 4:** Association between socio-demographics characteristics and viral shedding in stool.

Variables	Positive (%)	Negative (%)	χ^2^	P-value
**Age group (Years)**				
≤24	1 (9.1)	10 (90.9)	4.96	0.175
25–34	9 (24.3)	28 (75.7)		
35–44	9 (42.9)	12 (57.1)		
≥45	8 (36.4)	14 (63.6)		
**Gender**				
Male	23 (38.3)	37 (61.7)	6.33	0.012*
Female	4 (12.9)	27 (87.1)		
**Ethnicity**				
Yoruba	18 (26.9)	49 (73.1)	2.85	0.415
Hausa	3 (60.0)	2 (40.0)		
Igbo	5 (29.4)	12 (70.6)		
Others	1 (50.0)	1 (50.0)		
**Religion**				
Christian	23 (29.9)	54 (70.1)	0.01	0.922
Islam	4(28.6)	10(71.4)		
**Region**				
Semi-slum	12 (27.9)	31 (72.1)	0.12	0.727
Non-slum	15 (31.3)	33 (68.8)		
**Variable**				
Occupation			8.073	0.139
Healthcare Workers	5 (38.5%)	8 (61.5%)		
Civil Servants	9 (45%)	11 (55%)		
Traders & Unskilled Workers	4 (30.8%)	9 (69.2%)		
Unemployed	3 (18.8%)	13 (81.2%)		
Military/Armed Forces	3 (50%)	3 (50%)		
Customer Service Workers	3 (13%)	20 (87%)		

### Association between clinical parameters (Respiratory and other non-respiratory symptoms) and SARS-CoV-2 shedding in stools

Table 5 shows the association between clinical parameters and SARS-CoV-2 Viral Shedding in Stools. Higher proportion of respondents that had symptoms (41.2%) at admission shed the virus in their stool compared with those without symptoms (15%) at P-value<0.05. For instance, greater proportion of respondents with symptoms like fever (53.3%), fatigue (20.3%), headache (48.5%), catarrh, and loss of smell (52.4%) with P-values of <0.05 respectively, had the virus shed in their stools compared with those without the symptoms; fever (18%), fatigue (20.3%), headache (19%), and loss smell (22.9%).

**Table 5:** Association between clinical parameters (Respiratory and other non-respiratory symptoms) and SARs-CoV-2 viral shedding in stools.

Variables	Positive (%)	Negative (%)	χ2	P-value
**Symptoms at Admission**				
Yes	21(41.2)	30(58.8)	7.36	0.007*
No	6(15.0)	34(85.0)		
**Fever**				
Yes	16(53.3)	14(46.7)	12.01	0.001*
No	11(18.0)	50(82.0)		
**Fatigue / Body weakness**				
Yes	14(51.9)	13(48.1)	9.05	0.003*
No	13(20.3)	51(79.7)		
**Headache**				
Yes	16(48.5)	17(51.5)	8.78	0.003*
No	11(19)	47(81.0)		
**Cough**				
Yes	11(44)	14(56)	3.39	0.066
No	16(24.2)	50(75.8)		
**Catarrh**				
Yes	8(72.7)	3(27.3)	11.12	0.001*
No	19(23.8)	61(76.3)		
**Throat pain**				
Yes	3(30)	7(70)	0.00	0.981
No	24(29.6)	57(70.4)		
**Sneezing**				
Yes	4(57.1)	3(42.9)	2.74	0.098
No	23(27.4)	61(72.6)		
**Shortness of breath**				
Yes	2(33.3)	4(66.7)	0.04	0.839
No	25(29.4)	60(70.6)		
**Loss of smell**				
Yes	11(52.4)	10(47.6)	6.75	0.009*
No	16(22.9)	54(77.1)		
**Hiccups**				
Yes	1(25)	3(75)	0.04	0.834
No	26(29.9)	61(70.1)		

### Association between clinical parameters (Gastrointestinal) and SARS-CoV-2 shedding in stools

Table 6 shows the association between clinical parameters (abdominal) and SARS-CoV-2 shedding in stools. There was a high proportion of viral shedding in stool among participants with diarrhoea (50%) compared with those without diarrhoea (27.2%) at P-value>0.05. The proportion of viral shedding in stool was higher among respondents that had loss of taste (53.3%) compared with those that were without loss of taste (25%) at a P-value<0.05.

**Table 6:** Association between gastrointestinal symptoms and SARS-CoV-2 shedding in stools.

Variables	Positive	Negative	χ^2^	P-value
**Diarrhoea**				
Yes	5 (50)	5 (50)	2.23	0.136
No	22 (27.2)	59 (72.8)		
**Constipation**				
Yes	1 (100)	0 (0)	2.4	0.122
No	26 (28.9)	64 (71.1)		
**Anorexia/loss of appetite**				
Yes	3 (37.5)	5 (62.5)	0.26	0.612
No	24 (28.9)	59 (71.1)		
**Nausea**				
Yes	2 (50)	2 (50)	0.83	0.579
No	25 (28.7)	62 (71.3)		
**Vomiting**				
Yes	0 (0)	6 (100)	2.71	0.1
No	27 (31.8)	58 (68.2)		
**Abdominal pain**				
Yes	2 (22.2)	7 (77.8)	0.27	0.606
No	25 (30.5)	57 (69.7)		
**Loss of taste**				
Yes	8 (53.3)	7 (46.7)	4.82	0.028*
No	19 (25)	57 (75)		

### Association between co-morbidities and SARS-CoV-2 shedding in stools

Table 7 shows the association between co-morbidities and SARs-CoV-2 shedding in stools. The proportion of respondents with hypertension (47.4%), shedding the virus in their stools was higher at the point of admission compared with those without hypertension at a P-value <0.05 and this is significant.

**Table 7:** Association between co-morbidities and SARS-CoV-2 shedding stools.

Variables	Positive (%)	Negative (%)	χ2	P-value
**Hypertension**				
Yes	9 (47.4)	10 (52.6)	3.61	0.058
No	18 (25%)	54 (75.0)		
**Diabetes**				
Yes	1 (50)	1 (50)	0.41	0.525
No	26 (29.2)	63 (70.9)		
**Asthma**				
Yes	1 (33.3)	2 (66.7)	0.02	0.888
No	26 (29.5)	62 (70.5)		
**Peptic Ulcer Disease**				
Yes	3 (33.3)	6 (66.7)	0.06	0.8
No	24 (29.3)	58 (70.7)		
**Obesity (BMI>30)**				
Yes	0 (0)	2 (100)	0.86	0.578
No	27 (30.3)	62 (69.7)		

### Comparison between nasopharyngeal cycle threshold value and SARS CoV-2 shedding in stool among participants

Table 8 shows the comparison between nasopharyngeal shedding of SARS CoV-2 at the time of stool sampling. Low nasopharyngeal PCR CT-value was found to be significantly associated with viral shedding in their stool compared to moderate and high nasopharyngeal PCR CT-value (p-value = 0.08). Threshold value among the respondents that were negative (M = 31.64±6.13) and those that were positive for viral shedding in stools (M = 29.28±5.03); at t (89) = 1.77 P = 0.081.

**Table 8:** Comparison between nasopharyngeal cycle threshold and viral shedding in stool among participants.

NP PCR Ct-value	Viral Shedding in Stool		Total	X2 or t	P
	No of Positive	No of Negative			
31–40	1	12	13	9.69	0.008*
20–30	9	33	42		
<20	17	19	36		
**Total**	27	64	91		
Mean+/-SD	29.28 +/- 5.03	31.61 +/- 6.13	30.94 +/- 5.90	1.77	0.081

### Factor associated with SARS CoV-2 shedding in stools among participant (Multi-variate analysis)

Table 9 shows the factor associated with viral shedding in stools among respondents. Low and moderate viral load result was significantly associated with viral shedding in stool at p-value of 0.033 and 0.039 respectively.

**Table 9:** Factors associated with SARS-CoV-2 RNA shedding in stool of participants.

Variables	AOR	95%CI	P-value
**CT Value**			
High(ref)	1		
Moderate	0.28	0.08–0.94	0.039*
Low	0.08	0.01–0.80	0.033*
**Symptoms at admission**			
No(ref)	1		
Yes	1.63	0.31–8.48	0.559
**Fever**			
No(ref)	1		
Yes	1.07	0.22–5.14	0.932
**Fatigue/Body weakness**			
No(ref)	1		
Yes	1.11	0.22–5.53	0.897
**Headache**			
No(ref)	1		
Yes	1.98	0.49–7.97	0.338
**Loss of smell**			
No(ref)	1		
Yes	3.36	0.58–19.55	0.177
**Loss of taste**			
No(ref)	1		
Yes	1.17	0.20–6.90	0.859

## Discussion

The results of this study also reported that a large number of individuals infected with SARS-CoV-2, are residents living in non-slum areas who are civil servants; with almost half of the civil servants in this study shedding the SARS-CoV-2 in their stools. This lay credence to the belief among Nigerians that COVID-19 is a disease of the rich, but this is not true. According to the current finding, the reason for the higher percentage of individuals who lived in non-slum environment and were infected with COVID-19 might be due to the nature of their job, as civil servants, most especially, as they tend to work in confined environments and have several contact with the public [8]. This scenario aids the rapid transmission of this highly infectious virus. The study also showed that more than a third of healthcare workers and half of military personnel also have a more positive shed of the virus in their stool, although not statistically significant. As first responders in the control of COVID-19, this may be attributed to the higher risk of exposure among these individuals, and may be attributed to their constant contact with probable or confirmed patients [9], and the need for emergency outbreak response or law enforcement in the community [10].

The median age of the respondents was 37.78+/-14.41 years and majority of them were males. This finding revealed that younger population are also susceptible to COVID-19 infection. This is contrary to the belief that COVID 19 only affects the aged or elderly. A study by *Vaselli et al*. also reported a lower age category among patients infected with COVID 19 but more females than males and this could be due to difference in study locations in the United Kingdom [11].

Many of the patients infected with COVID-19 presented with fever and headaches, with the major respiratory symptoms being cough and loss of smell. A study by Li *et al*. also reported that 99% of patients present with fever and other common symptoms like loss of smell, myalgia and cough [12]. This finding is similar to that of this present study. This study revealed the less common symptoms as throat congestion and dyspnoea. According to this study, the most common symptom of COVID-19 is high fever [13], which majority of Nigerians always attribute to the onset of malaria. This may sometimes prevent early presentation of patients for medical care and timely health interventions; which may increase the severity of the viral disease. Hypertension and Peptic ulcer disease were the major co-morbidity found among participants which is in keeping with the finding by *Wang et al*. which showed that hypertension was a major cause of fatality among patients with COVID-19 and are more likely to develop serious symptoms with mortality rate of 6.0 [14]. Although there was no significant association between any of the co-morbid states assessed and shedding of the virus in stools. This indicates that shedding of the virus in stool was not influenced by the presence of any co-morbidity state. This finding is in agreement with finding by Tam *et al*. which showed that there was no association between co-morbidities and shedding of SARS CoV-2 in the stool of infected patients [15].

A significant finding was that male participants who shed the virus in stool were mainly within the age range of 35–44 years. This finding is consistent with *Zhang et al*., which highlighted that shedding of the virus in the stool occurred majority in male participants (60.2%) [16].

A significant percentage of patients who presented with fever (53.3%), fatigue/body weakness (51.9%), headache (48.5%), catarrh (72.7%) and loss of smell (52.4%) shed the virus in stool. There is dearth of literature on the association between these non-specific clinical symptoms and SARS-CoV-2 shedding in stool.

Viral shedding in stool was higher among respondents who had loss of taste. Loss of taste was recognized as a classical sign of COVID-19 in the early days and has also been linked with severity of the disease [5]. This is keeping with findings by Tam *et al*. which showed that SARS-CoV-2 gastrointestinal shedding is common and associated with gastrointestinal symptoms [15].

When the fluorescent signal exceeds the background fluorescence, the thermal cycles are defined as Cycle Threshold (CT). This is a semi-quantitative measure that helps in the broad categorization of viral genetic material in patient’s samples following testing by PCR. There is a higher proportion of participants with low CT-value shedding the virus in their stools compared with those having moderate and high CT-value.

This is in keeping with previous findings by Andrew fox-Lewis *et al*. [17], which revealed that a low CT-value of nasopharyngeal swab, which translated to a high viral load, was associated with shedding of SARS-CoV-2 in stool [18]. In daily practice, PCR CT-values were used as alternative markers for the amount of virus shed in a given sample and more importantly, to indicate the patient’s infectivity. However, the infective dose of the virus that is required to cause an infection is not yet known. Similarly, the persistence of the virus in patients through different stages of infection, or whether CT-values are associated with the viral load is yet to be reported.

### Limitations

Although viral RNA was detectable in the faeces of patients infected with SARS‐ CoV‐2, whether or not infectious live viruses could be excreted needs more studies. In addition, the study was limited to a small number of cases and a larger cohort would help provide better scientific understanding. Also, health care workers were separately captured in this study as only a demographic entity in the occupation variable; their particular risk of exposure to SARS-CoV-2 (such as their caring for or contact with confirmed COVID-19 patients, handling of infected biological samples, and their use or inadequate use of PPE) was not further explored at the time of this study. Finally, the cross-sectional nature of the study design does not provide evidence of causal relationship between the independent and dependent variables, as time sequence criteria cannot be fulfilled.

## Conclusion

This study revealed that about a third of the study participants shed SARS-CoV-2 RNA in their stool. Hence, gastrointestinal tract could be a source of transmission for SARS-CoV-2 in both symptomatic and asymptomatic patients. Also, findings from this study showed that low CT value of the nasopharyngeal swab from participants appeared to be related to shedding of the virus in the stool. This suggest the possibility of transmission through the faeco-oral route due to the presence of Viral RNA in stool. Further laboratory test analyses, such as viral culture on the stool samples is required to detect and demonstrate active viral elements in order to establish the infectivity potentials of the faeco-oral transmission among study participants.

## Supporting information

S1 Text (PDF)

S1 Data (SAV)
