# Supplementary material for: Clinical symptoms and faecal shedding of SARS-CoV-2 RNA among hospitalized COVID-19 patients: Implication for transmission
Source: PLOS Glob Public Health. 2024 Aug 28;4(8):e0003571. doi: 10.1371/journal.pgph.0003571 (PMC11357088; doi:10.1371/journal.pgph.0003571)
Supplement: S1 Text — (PDF) [file pgph.0003571.s001.PDF]

Performance Report COVD SARS-CoV-2 Molecular

Test Event 2021_2 Results Deadline 2021-07-28

Participant

Biorepository and Clinical Virology Laboratory

ID

NG1049AI

ACCEPTABLE	UNACCEPTABLE	NOT EVALUATED
100%	0%	0%

Program

SARS-CoV-2 Molecular

Order Code: COVD435

Subscription ID: 194797

ACCEPTABLE	UNACCEPTABLE	NOT EVALUATED
100%	0%	0%

Analytes

Analyte	Summary	ACCEPTABLE	UNACCEPTABLE	NOT EVALUATED
N1 gene Interpretation	5 ACCEPTABLE 0 UNACCEPTABLE 0 NOT EVALUATED.	100%	0%	0%
N2 gene Interpretation	5 ACCEPTABLE 0 UNACCEPTABLE 0 NOT EVALUATED.	100%	0%	0%
SARS-CoV-2 Interpretation	5 ACCEPTABLE 0 UNACCEPTABLE 0 NOT EVALUATED.	100%	0%	0%

oneworld ACCURACY™ | INTERNATIONAL

Performance Report

2021/Sep/09
Final

**Participant**	NG1049AI	Biorepository and Clinical Virology Laboratory
**Subscription ID**	194797	COVD435 SARS-CoV-2 Molecular
**Results Deadline**	2021/Jul/14	
**Accreditation**		Accredited by A2LA as compliant with ISO/IEC 17043:2010 under Oneworld Accuracy's scope of accreditation, number 4839.01.

Sample Condition

Date samples were received (YYYY/MMM/DD)	2021/Jul/15
Were the samples received in good condition?	Yes

Assay Da An Gene RNA/DNA Purific/Genechecker UF-300/DiagnoVital HS SARS-CoV-2 Real-Tim

N2 gene ct value

Sample	Run	Replicate 1			Replicate 2			Replicate 3			Peer Group Statistics					
		Result	Grade	SDI	Result	Grade	SDI	Result	Grade	SDI	Participants (n)	Results (n)	Statistical (n)	Mean	SD	CV(%)
A	1	36.26	⊖													
B	1	0.00	⊖													
C	1	33.37	⊖													
D	1	0.00	⊖													
E	1	33.69	⊖													

Peer Group Description

PG ID	PG Code	Test Process	Kit	Technique	Instrument Model

N1 gene ct value

Sample	Run	Replicate 1			Replicate 2			Replicate 3			Peer Group Statistics					
		Result	Grade	SDI	Result	Grade	SDI	Result	Grade	SDI	Participants (n)	Results (n)	Statistical (n)	Mean	SD	CV(%)
A	1	34.06	⊖													
B	1	0.00	⊖													
C	1	31.75	⊖													
D	1	0.00	⊖													
E	1	32.39	⊖													

Grade ✔ Acceptable ✖ Unacceptable ⊖ Not evaluated

Report was prepared and authorized by the ASPIRE unit of Oneworld Accuracy.
www.1wa.org
support@1wa.org | +1 800.665.2575 Canada+U.S. | +1 604.222.3900 Int. | fax
118 W 8th Ave Vancouver, BC, V5Y 1N2, Canada

Performance Report

**Participant**	NG1049AI	Biorepository and Clinical Virology Laboratory
**Subscription ID**	194797	COVD435 SARS-CoV-2 Molecular
**Results Deadline**	2021/Jul/14	
**Accreditation**	ACCREDITED Accredited by A2LA as compliant with ISO/IEC 17043:2010 under Oneworld Accuracy's scope of accreditation, number 4839.01.	

Assay Da An Gene RNA/DNA Purific/Genechecker UF-300/DiagnoVital HS SARS-CoV-2 Real-Tim

Peer Group Description

PG ID	PG Code	Test Process	Kit	Technique	Instrument Model

Grade Acceptable Unacceptable Not evaluated

Report was prepared and authorized by the ASPIRE unit of Oneworld Accuracy.
www.1wa.org
support@1wa.org | +1 800.665.2575 Canada+U.S. | +1 604.222.3900 Int. | fax
118 W 8th Ave Vancouver, BC, V5Y 1N2, Canada

Performance Report

**Participant**	NG1049AI	Biorepository and Clinical Virology Laboratory
**Subscription ID**	194797	COVD435 SARS-CoV-2 Molecular
**Results Deadline**	2021/Jul/14	
**Accreditation**	Accredited by A2LA as compliant with ISO/IEC 17043:2010 under Oneworld Accuracy's scope of accreditation, number 4839.01.	

Assay Da An Gene RNA/DNA Purific/Genechecker UF-300/DiagnoVital HS SARS-CoV-2 Real-Tim

N2 gene Interpretation

Sample	Result	Grade	Peer Group Counts: Participants (n)	Results (n)	Statistical (n)	Concordant with RR (n)	Concordant with RR (%)	Reference Results (RR)
A	Positive	Acceptable	1	1	1	1	100	Positive
B	Negative	Acceptable	1	1	1	1	100	Negative
C	Positive	Acceptable	1	1	1	1	100	Positive
D	Negative	Acceptable	1	1	1	1	100	Negative
E	Positive	Acceptable	1	1	1	1	100	Positive

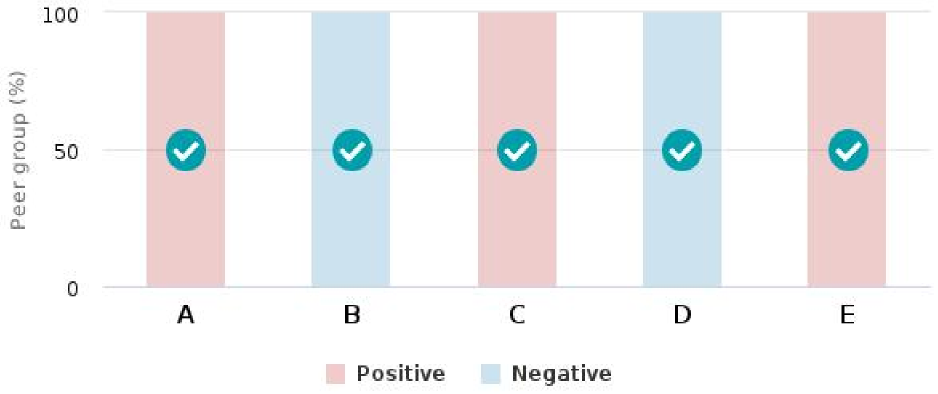

Peer Group Description

PG ID	PG Code	Test Process	Kit	Technique	Instrument Model
258080	AS3	Extraction	Da An Gene RNA/DNA Purification Kit		
		Amplification	Genechecker UF-300		

Grade: Acceptable, Unacceptable, Not evaluated

support@1wa.org | +1 800.665.2575 Canada+U.S. | +1 604.222.3900 Int. | fax
118 W 8th Ave Vancouver, BC, V5Y 1N2, Canada

Performance Report

Participant NG1049AI Biorepository and Clinical Virology Laboratory
Subscription ID 194797 COVD435 SARS-CoV-2 Molecular
Results Deadline 2021/Jul/14
Accreditation Accredited by A2LA as compliant with ISO/IEC 17043:2010 under Oneworld Accuracy's scope of accreditation, number 4839.01.

Assay Da An Gene RNA/DNA Purific/Genechecker UF-300/DiagnoVital HS SARS-CoV-2 Real-Tim

Peer Group Description

PG ID	PG Code	Test Process	Kit	Technique	Instrument Model
		Detection	DiagnoVital HS SARS-CoV-2 Real-Time PCR Kit		

N1 gene Interpretation

Sample	Result	Grade	Peer Group Counts					Reference Results (RR)
			Participants (n)	Results (n)	Statistical (n)	Concordant with RR (n)	Concordant with RR (%)	
A	Positive	Acceptable	1	1	1	1	100	Positive
B	Negative	Acceptable	1	1	1	1	100	Negative
C	Positive	Acceptable	1	1	1	1	100	Positive
D	Negative	Acceptable	1	1	1	1	100	Negative
E	Positive	Acceptable	1	1	1	1	100	Positive

Grade: Acceptable | Unacceptable | Not evaluated

Performance Report

**Participant**	NG1049AI	Biorepository and Clinical Virology Laboratory
**Subscription ID**	194797	COVD435 SARS-CoV-2 Molecular
**Results Deadline**	2021/Jul/14	
**Accreditation**	Accredited by A2LA as compliant with ISO/IEC 17043:2010 under Oneworld Accuracy's scope of accreditation, number 4839.01.	

Assay Da An Gene RNA/DNA Purific/Genechecker UF-300/DiagnoVital HS SARS-CoV-2 Real-Tim

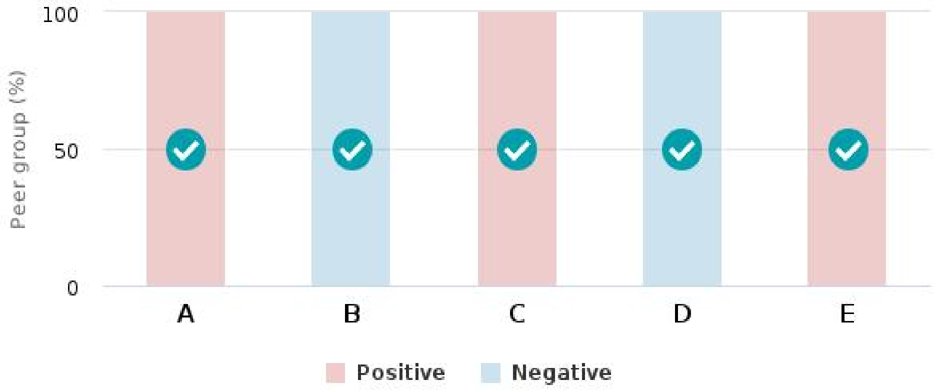

Peer Group Description

PG ID	PG Code	Test Process	Kit	Technique	Instrument Model
258080	AS3	Extraction	Da An Gene RNA/DNA Purification Kit		
		Amplification	Genechecker UF-300		
		Detection	DiagnoVital HS SARS-CoV-2 Real-Time PCR Kit		

Grade ✔ Acceptable ✖ Unacceptable ⊖ Not evaluated

support@1wa.org | +1 800.665.2575 Canada+U.S. | +1 604.222.3900 Int. | fax
118 W 8th Ave Vancouver, BC, V5Y 1N2, Canada

oneworld ACCURACY™ | INTERNATIONAL

Performance Report

2021/Sep/09
Final

**Participant**	NG1049AI	Biorepository and Clinical Virology Laboratory
**Subscription ID**	194797	COVD435 SARS-CoV-2 Molecular
**Results Deadline**	2021/Jul/14	
**Accreditation**	Accredited by A2LA as compliant with ISO/IEC 17043:2010 under Oneworld Accuracy's scope of accreditation, number 4839.01.	

SARS-CoV-2 Interpretation

Sample	Result	Grade	Peer Group Counts: Participants (n)	Results (n)	Statistical (n)	Concordant with RR (n)	Concordant with RR (%)	Reference Results (RR)
A	Positive	Acceptable	220	220	220	214	97	Positive
B	Negative	Acceptable	220	220	220	213	97	Negative
						2	1	Invalid
C	Positive	Acceptable	220	220	220	211	96	Positive
D	Negative	Acceptable	220	220	220	217	99	Negative
E	Positive	Acceptable	220	220	220	210	95	Positive
						1	0.5	Presumptive Positive
						1	0.5	Equivocal/Grey Zone

Peer Group Description

PG ID	PG Code	Test Process	Kit	Technique	Instrument Model
0	AR	All Results			

Grade: Acceptable | Unacceptable | Not evaluated

support@1wa.org | +1 800.665.2575 Canada+U.S. | +1 604.222.3900 Int. | fax
118 W 8th Ave Vancouver, BC, V5Y 1N2, Canada

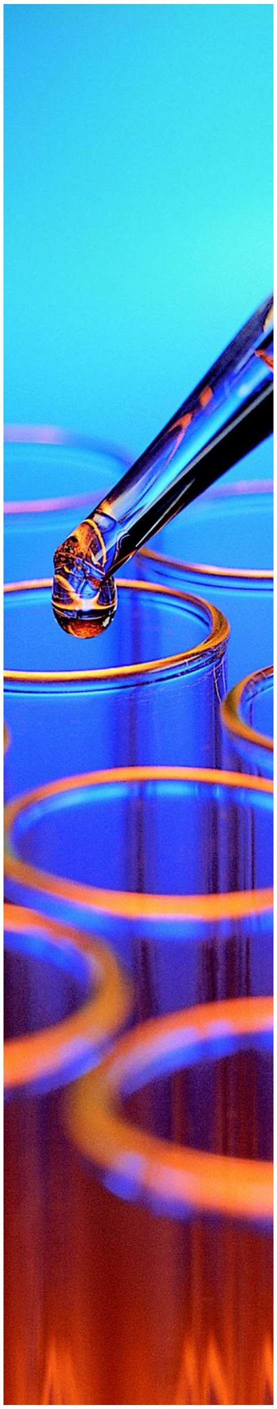

Test Event Summary

SARS-CoV-2 Molecular

COVD435

Status: Final<br><br>Version #:1.0	Issue Date: 2021-09-09<br><br>Published Results Deadline Date: 2021-07-14<br>Actual Results Deadline Date: 2021-08-11

Report prepared and authorized by the ASPIRE unit of Oneworld Accuracy.

Oneworld Accuracy

Oneworld Accuracy is a social enterprise group headquartered in Vancouver, British Columbia, Canada. We are guided by the conviction that accurate medical tests are a fundamental human right for all people in all countries as they enable doctors to make proper decisions so patients receive proper care.

Our Mission: To achieve universal testing accuracy for improved healthcare for all people.

Copyright

The information and data included in this report are the intellectual property of Oneworld Accuracy Inc. This report and its contents may not be reproduced, in whole or in part, for any purpose without the written permission of Oneworld Accuracy; nor may they be used in any type of advertising or sales promotion.

Confidentiality Commitment

The identity of participants in EQA programs shall be confidential and known only to persons involved in the operation of the programs, unless the participant waives confidentiality.

All information supplied by a participant to Oneworld Accuracy shall be treated as confidential.

When an interested party requires the test results to be directly provided by Oneworld Accuracy Inc, the participants shall be made aware of the arrangement in advance of participation.

Activities Subcontracted

Oneworld Accuracy subcontracts the following activities for all EQA programs: manufacturing of EQA samples, testing samples for homogeneity and stability and delivery of EQA samples to participants.

All Oneworld Accuracy subcontractors are thoroughly reviewed and evaluated on a regular basis. Oneworld Accuracy seeks ISO certification from all subcontractors wherever possible.

About the EQA Program

The SARS-CoV-2 Molecular - COVD435 program includes 5 sample(s) consisting of FLOQSwab® distributed in 3 shipment(s) for the 3 test event(s) in the year. The current event opened on 2021-06-23 and had a published results deadline of 2021-07-14 (actual results deadline 2021-08-11).

If storage was necessary, samples were stored at 2-30°C | 35-86°F from the time of receipt until dispatching to the participants. All shipments were packed with the appropriate amount of stabilizers, if necessary, to ensure the required temperature was maintained throughout the regular delivery time. Any exceptions were recorded and results reviewed at the time of evaluation.

Statistical Analysis

For quantitatively reported analytes Oneworld Accuracy applies certain sections from **ISO 13528:2015 - Statistical methods for use in proficiency testing by interlaboratory comparison** for calculating the robust mean and standard deviation for our evaluations. For qualitatively reported analytes, evaluation is based on consensus between participants and/or concordance between reported results and reference values as provided by the manufacturer, consultants, or consultant laboratories. If the analyte is reported quantitatively, then one of the following evaluation methods would apply:

1. If the assigned value is derived from the peer group mean -

The evaluation criteria may be peer group mean +/- SD, percentage (%), or an absolute value. Peer group mean is the robust mean of the results included in a statistically valid peer group created with like instruments, reagents, or methods, once statistical outliers are removed. The upper and lower limits of the acceptable range are determined from that described in the evaluation criteria as shown in the performance report.

The peer groups are shown in the performance report. The different peer groups and their associated statistics are shown in the Participation Statistics for the program and available for download from the Oneworld Accuracy website.

2. If the assigned value is derived from a reference method -

The evaluation criteria would be Reference value +/- percentage (%). Reference values can be traceable values as provided by accredited reference laboratories using reference methods, or be gravimetrically 'weighted-in'. The upper and lower limits of the acceptable range are determined from that described in the evaluation criteria as shown in the performance report.

Some programs apply Multiple Assessment Criteria (MAC) as the grading system. Depending on the percentage difference between the reference value and the reported result, results can be graded as Unacceptable, Minimal, Desirable, and Optimal.

Click here for an interpretation guide for our performance reports, as well as a troubleshooting guide for EQA results

www.1wa.org
support@1wa.org | +800.665.2575 | +800.665.5949 fax
118 W 8th Ave Vancouver, BC, V5Y 1N2, Canada

Comments and Observations

For the test event (results deadline 2021-07-14, actual results deadline 2021-08-11), there were 3395 expected results. There were 3393 results submitted providing a participation rate of 99.94% collaboration wide.

Evaluation can be conducted with additional data from a different program format, thus the participation counts mentioned here might be different than those seen on performance reports and participation statistics reports.

Participants submitted results online using OASYS with a unique identifier assigned for each participant.

Of the results that were submitted, the percentage of results that were Acceptable (ACC) was 95.82%, Unacceptable (UNACC) 2.94% and Not-Evaluated (NE) 1.24%.

The percentage of Acceptable (ACC), Unacceptable (UNACC) and Not-Evaluated (NE) results by sample and analyte is shown below.

For samples with high NE (Not Evaluated) rates, it could be due to lack of peer group statistics or an evaluation decision being applied. Please review the participation statistics report or the performance report for additional information or commentary.

Table A: Performance by Analyte

Analyte	Sample	% ACC	% UNACC	% NE
ORF1ab gene Interpretation	A	93.00	6.00	1.00
	B	99.00	0.00	1.00
	C	94.00	5.00	1.00
	D	97.00	2.00	1.00
	E	97.00	2.00	1.00
SARS-CoV-2 Interpretation	A	97.27	2.73	0.00
	B	97.73	2.27	0.00
	C	95.91	4.09	0.00
	D	98.64	1.36	0.00
	E	96.36	3.64	0.00
N gene Intepretation	A	93.41	6.59	0.00
	B	96.70	3.30	0.00
	C	92.31	7.69	0.00
	D	97.80	2.20	0.00
	E	93.41	6.59	0.00
S gene Interpretation	A	100.00	0.00	0.00
	B	100.00	0.00	0.00
	C	95.45	4.55	0.00
	D	95.45	4.55	0.00
	E	95.45	4.55	0.00
RdRp gene Interpretation	A	100.00	0.00	0.00
	B	100.00	0.00	0.00
	C	87.50	12.50	0.00
	D	100.00	0.00	0.00
	E	95.83	4.17	0.00
E gene Interpretation	A	98.25	1.75	0.00
	B	99.13	0.87	0.00
	C	95.65	4.35	0.00
	D	99.13	0.00	0.87
	E	95.65	4.35	0.00
N2 gene Interpretation	A	98.63	1.37	0.00
	B	100.00	0.00	0.00
	C	98.65	1.35	0.00
	D	100.00	0.00	0.00
	E	97.30	2.70	0.00
N1 gene Interpretation	A	83.33	16.67	0.00
	B	100.00	0.00	0.00
	C	100.00	0.00	0.00
	D	100.00	0.00	0.00

www.1wa.org
support@1wa.org | +800.665.2575 | +800.665.5949 fax
118 W 8th Ave Vancouver, BC, V5Y 1N2, Canada

Table A: Performance by Analyte

Analyte	Sample	% ACC	% UNACC	% NE
N1 gene Interpretation	E	83.33	16.67	0.00
RdRp/N gene Interpretation	A	100.00	0.00	0.00
	B	94.74	5.26	0.00
	C	100.00	0.00	0.00
	D	100.00	0.00	0.00
	E	100.00	0.00	0.00
ORF1ab gene Reactivity	A	81.82	9.09	9.09
	B	90.91	0.00	9.09
	C	90.91	0.00	9.09
	D	90.91	0.00	9.09
	E	90.91	0.00	9.09
ORF8 gene Interpretation	A	40.00	20.00	40.00
	C	40.00	20.00	40.00
	E	60.00	0.00	40.00
ORF8 gene Reactivity	A	40.00	20.00	40.00
	C	40.00	20.00	40.00
	E	60.00	0.00	40.00
RdRp/S gene Interpretation	A	100.00	0.00	0.00
	B	100.00	0.00	0.00
	C	100.00	0.00	0.00
	D	100.00	0.00	0.00
	E	100.00	0.00	0.00
E / N gene Interpretation	A	100.00	0.00	0.00
	B	100.00	0.00	0.00
	C	100.00	0.00	0.00
	D	100.00	0.00	0.00
	E	100.00	0.00	0.00

Note – Samples and/or analytes that are not evaluated are not displayed. Reasons can include lack of peer group statistics for evaluation and evaluation decisions such as lack of consensus between results. Refer to participation statistics reports and/or performance reports for additional information/commentary. Samples and/or analytes in which there are no submitted results are also not displayed.

Analytical problem(s) were submitted by participants who were unable to perform testing, or to get a result for a particular analyte or sample. The percentage of submitted results that had an analytical problem entered was 0.91%, and they were not evaluated (NE). The breakdown of analytical problem(s) submitted is shown below.

Table B: Analytical Problem(s)

Analytical Phase	Analytical Problem	%
**Other**	Not Measured	0.88
	Not Determinable	0.03

If possible and applicable, before each test event, participants should ensure they have appropriate reagents/consumables and instruments that are up-to-date with maintenance procedures, to prevent entry of a pre-analytical AP code. AP codes regarding linear/detection limits can indicate analytical issues concerning validity of a method’s measurement range, or samples that have analyte concentrations exceeding a method's detection ranges.
